# Effects of TROL Presequence Mutagenesis on Its Import and Dual Localization in Chloroplasts

**DOI:** 10.3390/ijms19020569

**Published:** 2018-02-14

**Authors:** Lea Vojta, Andrea Čuletić, Hrvoje Fulgosi

**Affiliations:** Laboratory for Plant Molecular Biology and Biotechnology, Division of Molecular Biology, Ruđer Bošković Institute, Bijenička cesta 54, 10000 Zagreb, Croatia; aculetic@bot.uni-kiel.de (A.Č.); fulgosi@irb.hr (H.F.)

**Keywords:** TROL, protein import, chloroplasts, dual localization, ATP, inner envelope membrane, thylakoids

## Abstract

Thylakoid rhodanase-like protein (TROL) is involved in the final step of photosynthetic electron transport from ferredoxin to ferredoxin: NADP^+^ oxidoreductase (FNR). TROL is located in two distinct chloroplast compartments—in the inner envelope of chloroplasts, in its precursor form; and in the thylakoid membranes, in its fully processed form. Its role in the inner envelope, as well as the determinants for its differential localization, have not been resolved yet. In this work we created six N-terminal amino acid substitutions surrounding the predicted processing site in the presequence of TROL in order to obtain a construct whose import is affected or localization limited to a single intrachloroplastic site. By using in vitro transcription and translation and subsequent protein import methods, we found that a single amino acid exchange in the presequence, Ala67 to Ile67 interferes with processing in the stroma and directs the whole pool of in vitro translated TROL to the inner envelope of chloroplasts. This result opens up the possibility of studying the role of TROL in the chloroplast inner envelope as well as possible consequence/s of its absence from the thylakoids.

## 1. Introduction

TROL (thylakoid rhodanase-like protein, At4g01050) is an integral membrane component of non-appressed thylakoid membranes, responsible for the anchoring of FNR (ferredoxin:NADP^+^ oxidoreductase) [1]. This 66 kDa protein is firmly attached to the membrane by its two transmembrane helices, resisting extraction by high salt, urea, or high pH treatments [1]. The N-terminus of TROL consists of a presequence that directs the protein to chloroplasts. Two predicted transmembrane helices surround the centrally positioned inactive rhodanase-like domain, RHO, which is orientated towards the thylakoid lumen. The C-terminus of the protein resides in the cytosol and consists of a single hydrophobic FNR-binding region, ITEP (highly conserved module of TROL necessary for establishing high-affinity interaction with FNR), and a region upstream of the ITEP domain, designated PEPE (Pro-Val-Pro repeat-rich region), which is followed by a possible PVP hinge, proposed to introduce flexibility to the FNR-binding region. In our previous research we proposed that the TROL–FNR interaction is dynamic [2,3], in which binding and release of FNR from TROL can regulate the flow of photosynthetic electrons before the pseudo-cyclic electron transfer pathway becomes activated [4]. By studying Arabidopsis *trol* mutants we proposed that the TROL–FNR interaction is the bifurcating point between electron-conserving and electron-dissipating pathways [4].

In addition to being mainly located at the stroma thylakoids, in its fully processed form of 66 kDa, TROL can also be found embedded in the chloroplast inner envelope membrane (IM) in its non-processed form (70 kDa), which indicates a possible role in the electron transfer chain specific for this membrane [1]. This dual localization has been proven by the proteome analysis of chloroplast envelopes and thylakoid membranes [5] and was proposed to be dependent on the NADP^+^/NADPH ratio in the chloroplasts, as shown for the shuttling of the Tic62 protein [6]. Although there were attempts to investigate the structure and the function of TROL as the FNR anchor [1,7,8,9] its role in the IM remains undefined.

TROL is a nuclear-encoded protein, synthesized with a cleavable N-terminal presequence that targets the protein to and across the chloroplast envelope. During chloroplast protein import, the targeting sequences are sequentially decoded resulting in localization of the polypeptide to the appropriate organelle subcompartment [10]. Since TROL has been found both in the IM and the thylakoids [1,5], the determinants for this dual localization were the subject of investigation in this research.

Most of the IM proteins are directed to the general import pathway (Toc and Tic complex) through their cleavable transit peptides on their way to the chloroplasts. Some preproteins are released from the translocon at the level of the IM, as instructed by a hydrophobic stop-transfer signal in their sequence [11]. Others are first targeted to the stroma by their stroma-targeting presequence, and their processed mature form is subsequently re-exported into the IM, by so-called conservative sorting [11]. Proteins targeted to the thylakoid membrane require dual targeting signals that direct their import across the chloroplast envelope membranes and subsequent transport to the thylakoids. Stroma-targeting domains of preproteins are recognized and removed by the stromal processing peptidase (SPP) and lumen-targeting domain by a second processing protease [10,12]. A single bipartite transit sequence can carry the information for targeting to the thylakoids, with the stromal targeting domain located at the N-terminal region and the thylakoid lumen targeting domain at the C-terminal region of the presequence [10,13]. Preplastocyanin (prePC), and the subunits of the oxygen evolving complex (preOE16, preOE23, and preOE33) are configured as described. Integral membrane proteins, such as the precursor to the light-harvesting chlorophyll a/b binding protein (preLHCP) and the precursor to the 20-kDa subunit of the CP24 complex [13], contain information only for envelope transport within the presequence. The signals for targeting of these proteins to the thylakoids seem to reside within the primary structure of the mature polypeptide [14]. Since a single targeting domain is predicted for the transit sequence of TROL, this protein might belong to the latter group.

Targeting sequences are of various sizes, from 30 to 120 amino acids, and contain a high number of hydroxylated residues, but lacking acidic ones. The N-terminal 10–15 residues are devoid of Gly, Pro, and charged residues, a variable middle region is rich in Ser, Thr, Lys, and Arg, lacking acidic residues; and a C-terminal proteolytic processing site [15]. It contains a loosely conserved Ile/Val-x-Ala/Cys-Ala motif, recognized by SPP. 

It has been postulated that chloroplast-localized proteins have various energy requirements for their import, according to their localization. ATP in the intermembrane space, usually less than 50 µM, drives the transport of the precursor across the outer envelope membrane [16,17], and import through the IM into the stroma progresses if the ATP concentration is higher than 100 µM [18,19]. This ATP is needed for the action of molecular chaperones in the stroma, which provide the driving force to complete import into the organelle [20]. Proteins localized in the same organellar compartment might have different energy needs for their import, depending on the import pathway they use [17]. Upon entering the stroma, the transit sequence is removed by SPP [20]. Further import into thylakoids requires additional translocators and energy demand. Four different mechanisms are known to target proteins from the stroma to the thylakoids [20,21,22]. 

The aim of this work was to introduce mutations in the presequence of the TROL protein, around the processing site, in an attempt to obtain a construct that would direct TROL to a single sub-chloroplast compartment, namely to a single membrane: either the inner envelope or the thylakoids. In addition, we wanted to characterize TROL import properties and requirements in more detail.

## 2. Results

**Figure 1:**
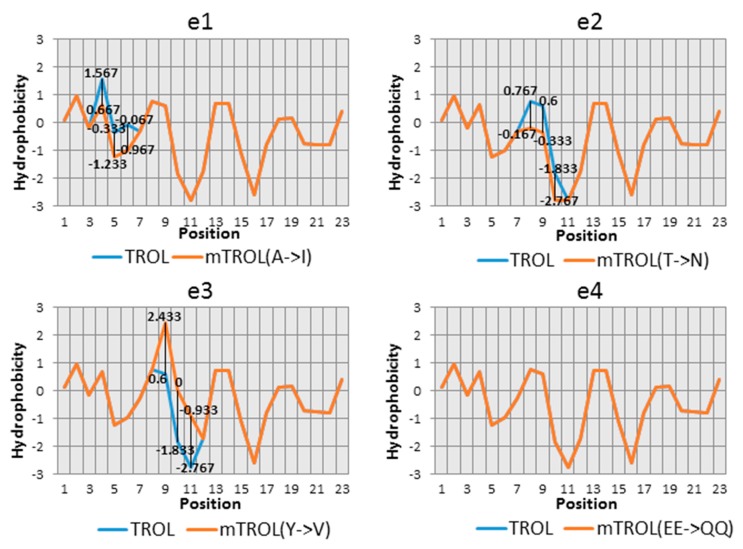

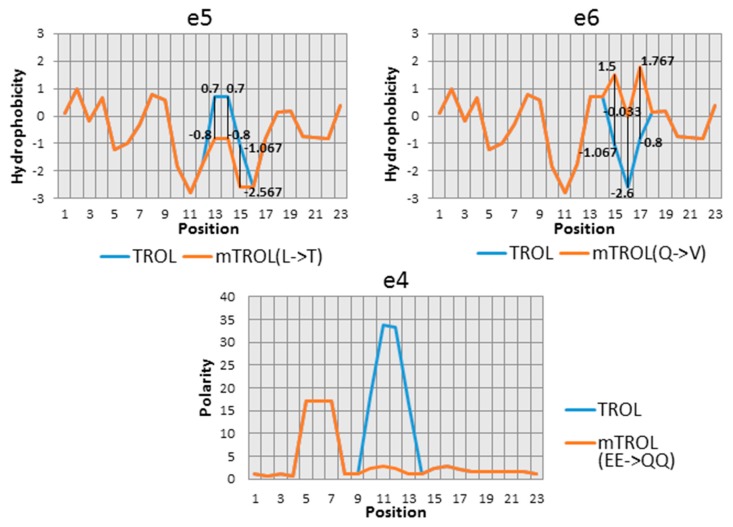
Kyte and Doolittle plots representing hydrophobicity change as a consequence of amino acid substitution/s in the TROL presequence [23]. Amino acids 67–78 of the partially conserved N-terminal part of the presequence around the predicted transit peptide cleavage site, AKSLTYEEALQQ, were substituted as follows: e1: 67Ala→67Ile, e2: 71Thr→71Asn, e3: 72Tyr→72Val, e4:73Glu74Glu→73Gln74Gln, e5: 76Leu→76Thr, e6:78Gln→78Val. For the e6 substitution, a polarity check according to Zimmerman was performed [24].

After amino acid comparison between TROL from *A. thaliana* and other vascular plants, the N-terminal conserved region was determined around the SPP cleavage site. In this conserved region, AKSLTYEEALQQ (aa 67–78), we have chosen six potentially significant amino acids that could influence the import and localization of protein TROL in chloroplasts. Changes made to the presequece were as following: e1: 67Ala→67Ile, e2: 71Thr→71Asn, e3: 72Tyr→72Val, e4: 73Glu74Glu→73Gln74Gln, e5: 76Leu→76Thr, e6: 78Gln→78Val (Table 1). Hydrophobicity was checked for each amino acid substitution, according to Kyte and Doolittle [23] (Figure 1). For substitutions 73Glu74Glu→73Gln74Gln, the exchange of polarity has been compared according to Zimmerman et al. [24] (Figure 1). After the mutations were introduced into the gene *At4g01050*, constructs in the pZL1 vector were checked by DNA sequencing.

**Figure 2:**
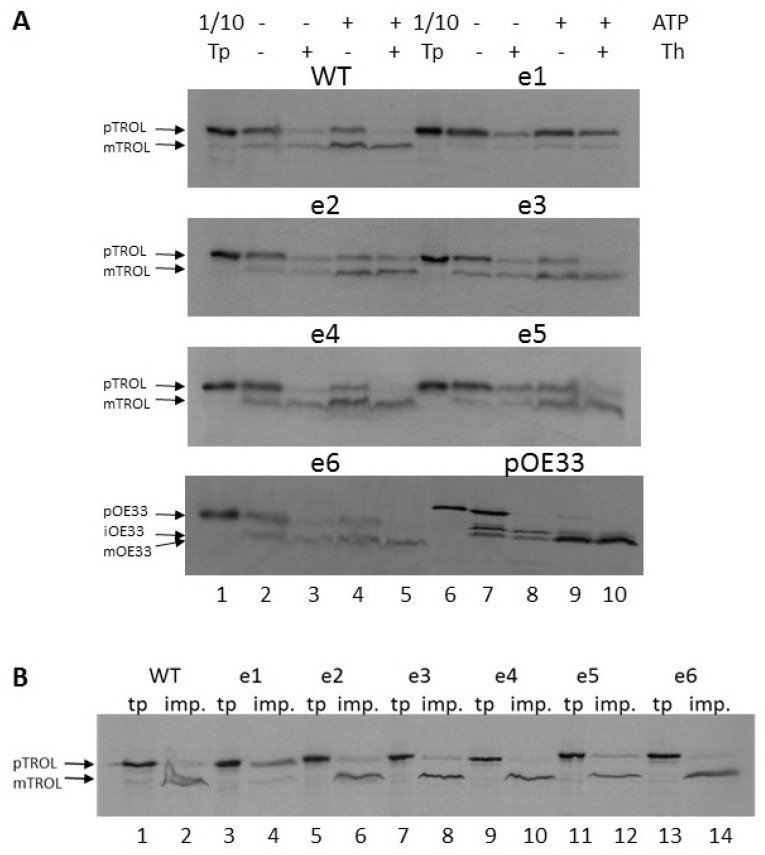
(**A**) Wt TROL and its presequence mutants e1–e6 are imported into pea chloroplasts. In vitro synthesized [^35^S]-TROL and e1–e6, as well as the control protein pOE33 from thylakoid lumen were incubated with isolated intact chloroplasts at 25 °C for 20 min, in a standard import reaction containing 3 mM ATP (lanes 4, 5, 9, and 10) or without ATP (lanes 2, 3, 7, and 8). After import, samples were re-isolated on a Percoll cushion and treated with 0.5 µg thermolysin (Th) per µg chlorophyll (lanes 3, 5, 8, and 10). Untreated samples are shown in lanes 2, 4, 7, and 9. The results were analyzed by SDS⁄PAGE. Lanes 1 and 6 represent 10% of the translation product (Tp) used for the import reactions. The positions of pTROL, mTROL, pOE33, iOE33, and mOE33 are indicated by arrows. (**B**) Comparison of import of TROL and its presequence mutants e1–e6 into chloroplasts. In vitro synthesized [^35^S]-TROL and e1–e6 were incubated with isolated intact chloroplasts at 25 °C for 20 min, in a standard import reaction containing 3 mM ATP. After import, samples were re-isolated on a Percoll cushion and treated with 0.5 µg thermolysin (Th) per µg chlorophyll (lanes 2, 4, 6, 8, 10, 12, and 14). The results were analyzed by SDS⁄PAGE. Lanes 1, 3, 5, 7, 9, 11, and 13 represent 10% of the respective translation product. The positions of pTROL and mTROL are indicated by arrows.

The wild type precursor of TROL and its e1–e6 presequence mutants were labeled with [^35^S]-methionine during in vitro translation and imported into isolated intact pea chloroplasts under various conditions. First, the standard import experiment was performed, in the absence or the presence of externally added 3 mM ATP. After a 20-min long import at 25 °C, chloroplasts were re-isolated on a Percoll cushion and treated with the protease thermolysin, to distinguish precursors loosely bound to the chloroplast envelope (early intermediates) from the firmly incorporated ones [17,18]. We observed that TROL requires ATP for successful processing and its import into the thylakoid membrane. After import with ATP, the majority of TROL was found in thylakoids, in its fully processed form, and just a small portion was located in the chloroplast envelope, in its precursor form, protected from thermolysin (Figure 2a, WT, lane 5). Presequence mutants e2, e3, e4, and e6 showed identical import behaviour as the wild type TROL (Figure 2a,b). In contrast, mutant e1 locates almost exclusively to the envelope membrane (Figure 2a, e1, lane 10, Figure 2b, lane 4). Only a very small portion was processed to the mature form and transported to the thylakoids. It seems that just a single amino acid substitution in the TROL presequence (67Ala→67Ile) results in almost exclusive envelope localization (Figure 2a,b). Presequence mutant e5, although having very similar import properties to the wild-type TROL, seems to have a slightly increased portion of envelope-bound form after import into chloroplasts (Figure 2a, e5, lanes 9 and 10, Figure 2b, lane 12).

**Figure 3:**
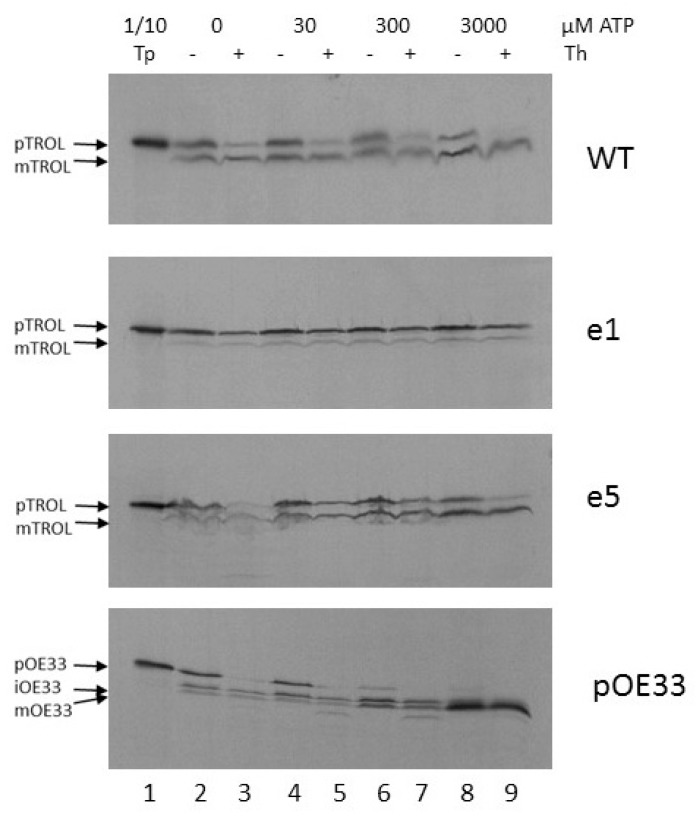
Energy requirement for import of wt TROL and its presequence mutants e1 and e5 into pea chloroplasts. Import into intact pea chloroplasts was performed under standard conditions, by incubating in vitro synthesized [^35^S]-TROL, e1, e5, and control protein pOE33 from thylakoid lumen with chloroplasts corresponding to 20 µg chlorophyll at 25 °C. ATP-scale import into intact pea chloroplasts was performed using increasing concentrations of ATP from 0 to 3000 µM. After import, chloroplasts were re-isolated on a Percoll cushion and samples were treated with 0.5 µg thermolysin (Th) per µg chlorophyll (lanes 3, 5, 7 and 9). Untreated samples are shown in lanes 2, 4, 6, and 8. The results were analyzed by SDS/PAGE. The respective precursor, intermediate, and mature forms are indicated by arrow heads. Lane 1 represents 1/10 of the translation product (Tp) used for the import reaction.

Further, we wanted to investigate the energy requirements for the import of TROL and its presequence mutants e1 and e5. After isolating chloroplasts from peas grown in the dark for at least 8 h, and incubating isolated intact chloroplasts in the dark and on ice to minimize internal ATP production, we added 0, 30, 300, or 3000 µM of external ATP to the import reaction. After 20 min. import at 25 °C and subsequent chloroplast re-isolation, we observed that even the smallest amount of ATP enabled the import of TROL into the thylakoids to some extent (Figure 3, lanes 3 and 5). Additional ATP resulted in a slight increase in the quantity of processed protein, while at the same time the portion of precursor form, located in the inner envelope, decreased (Figure 3, lanes 7 and 9). Mutant e5 seems to require higher amounts of external ATP to complete the import (Figure 3, e5). Also, there is more envelope form present in this mutant (Figure 3, lanes 5, 7, 9). Mutant e1 localizes almost exclusively to the chloroplast inner envelope, needing some external ATP (30–300 µM) for complete protection from thermolysin, as required for inner envelope incorporation (Figure 3, e1). As a control, oxygen evolving complex protein of 33 kDa (pOE33) was imported along with the TROL constructs. This is a well-characterized protein which is localized to the thylakoid lumen and forms a soluble translocation intermediate in the stroma. It uses the general import pathway into the chloroplasts and is therefore a suitable control for TROL import experiments. It seems that pOE33 requires more ATP to complete its import. With the lowest ATP concentration, stromal intermediate iOE33 is visible, and import seems to be complete after the addition of more than 300 µM ATP (Figure 3, pOE33). The higher energy need for pOE33 import results probably from its two processing events (in the stroma and in the thylakoid lumen) and its luminal localization.

**Figure 4:**
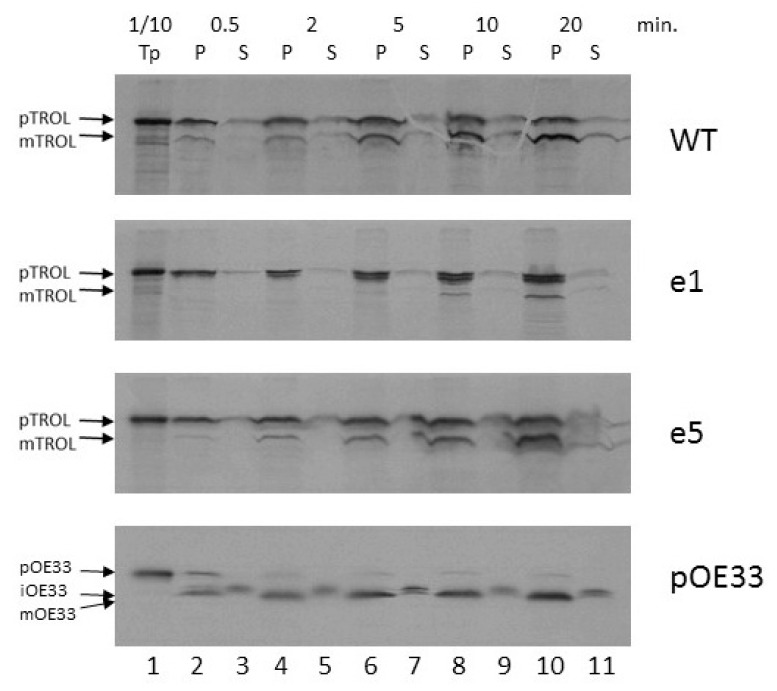
Time-scale import of wt TROL and e1 and e5 presequence mutants into intact pea chloroplasts. Radioactively labeled TROL, e1, e5, and control protein pOE33 were imported using increasing times in standard import reactions at 25 °C in the presence of 3 mM ATP. Import was performed for 0.5 (lanes 2 and 3), 2 (lanes 4 and 5), 5 (lanes 6 and 7), 10 (lanes 8 and 9), or 20 min (lanes 10 and 11). After import chloroplasts were separated into the pellet (P, lanes 2, 4, 6, 8, and 10) and soluble (S, lanes 3, 5, 7, 9, and 11) fractions. Lane 1 indicates 1/10 of the respective translation product (Tp) used for the import reaction. Precursor (p), intermediate (i), and mature (m) forms of TROL, e1, e5, and OE33 are indicated by arrows.

We also wanted to explore how the import of TROL and e1 and e5 mutants proceeds on a temporal scale. Radioactively labeled precursors were added to the standard import reaction including 3 mM ATP and chloroplasts corresponding to 20 µg chlorophyll. Imports were performed for 0.5, 2, 5, 10, and 20 min at 25 °C. All samples were subsequently re-isolated on Percoll cushion, treated with thermolysin, lysed, and separated into membrane (P) and soluble fractions (S). After the analysis of radioactive signals on films, it was clearly visible that as time advances the amount of proteins in the pellet fractions increases for all tested precursors (Figure 4). Between 5 and 10 min of import are needed for around 50% of TROL import to be accomplished. For complete import, more than 10 min were required (Figure 4, lane 10). For the e1 mutant, the portion of imported protein that locates to the IM visibly increases with time, while the amount of mature, thylakoid-localized form starts to appear after 10 min. of import (Figure 4, lane 8). In this experiment, mutant e5 showed nearly identical properties to wt TROL.

**Figure 5:**
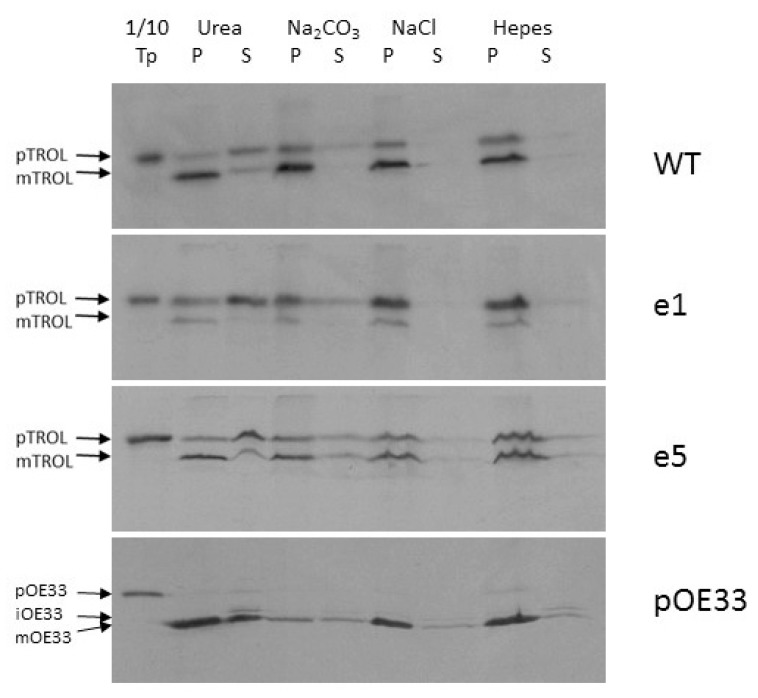
Extraction of wt TROL and its presequence mutants e1 and e5 from the membranes. In vitro synthesized [^35^S]-TROL, e1, e5, and control protein pOE33 were incubated with isolated intact chloroplasts at 25 °C for 20 min, in a standard import reaction containing 3 mM ATP. Subsequent to import, chloroplasts were re-isolated, washed, and separated into membrane and soluble fractions. Isolated membranes, containing imported proteins, were treated with 6 M Urea in 10 mM HEPES/KOH pH 7.6 (lanes 2 and 3), 0.1 M Na_2_CO_3_ pH 11.5 (lanes 4 and 5), or 1 M NaCl (lanes 6 and 7). All incubations were performed for 20 min on RT. As a control, membranes were incubated solely in 10 mM HEPES/KOH pH 7.6 for 30 min on ice (lanes 8 and 9). Afterwards, samples were centrifuged at 265,000× *g* for 10 min at 4 °C, and both pellets (P, lanes 2, 4, 6, and 8) and the supernatants (S, lanes 3, 5, 7, and 9) were analyzed by SDS–PAGE and by exposure on X-ray films. Lane 1 represents 10% of the respective translation product (Tp) used for the import reactions. The positions of pTROL, mTROL, pOE33, iOE33, and mOE33 are indicated by arrows.

Finally, we investigated to what extent is TROL, imported both to the IM and the thylakoids, associated with the membranes. The strength and the nature of this association was tested by applying either 6 M Urea, 0.1 M Na_2_CO_3_ pH 11.5 for the separation of integral from peripheral membrane proteins, or 1 M NaCl, which decreases electrostatic interactions between proteins and charged lipids. 6 M Urea only partially extracted imported TROL from the membranes, mostly its envelope-located portion, while the mature forms from thylakoid membranes remained almost fully intact (Figure 5, lanes 2 and 3). Treatment of the envelopes with Na_2_CO_3_ at pH 11.5 converts membrane vesicles to sheets and disrupts protein–protein interactions, while protein–lipid interactions remain and the bilayer is otherwise intact. In this way we could determine if an integral membrane protein has achieved stable insertion into the bilayer. Carbonate treatment extracted only a very small portion of the tested proteins from the membrane, indicating that TROL and the tested mutants are strongly attached to the lipid bilayer (Figure 5, lanes 4 and 5). High salt did not extract TROL from the membranes (Figure 5, lanes 6 and 7), indicating strong ionic interactions between TROL and the membranes.

## 3. Discussion

In the N-terminal conserved region of TROL, around the SPP cleavage site, we have chosen six potentially significant amino acids for its import and localization in chloroplasts. Selection of the amino acids was largely based on their hydrophobic or hydrophilic character, which we tried to change by substitutions, in an attempt to interfere with stromal processing. The structure and influence of the neighboring amino acids have also been taken into consideration. We have chosen those amino acids whose neighboring hydrophobic/hydrophilic signals were as indiscernible as possible. In the e1 mutation, Ala67 was changed to Ile. Ala67 is a part of the AXS motif, predicted to represent a signal for cleavage of the presequence by the SPP. Ile is similar in nature to Ala, but contains three more methyl groups that make it more hydrophobic. In the e2 mutation, neutral and polar Thr71 was changed to hydrophobic Asn. The corresponding Kyte and Doolittle plot [23] resulted in a neutral to slightly hydrophilic character of the changed amino acid site. In e3, by Tyr72 to Val exchange, strong hydrophilicity was substituted by medium to strong hydrophobicity. In this example the structure has also been changed by removing the cyclic ring in Tyr, which could additionally influence the hydrophobicity. In e4, where Glu73 and Glu74 were changed to two Gln, hydrophobicity remained the same, but the loss of polarity (according to Zimmerman et al. [24]) could influence protein sorting. In e5 hydrophobic Leu76 was exchanged for neutral Thr, leading to a more hydrophilic character. In e6 Gln78 to Val exchange leads to a large hydrophilicity loss and change to hydrophobicity. After successfully introduced mutations into TROL presequence, constructs e1–e6, as well as the wild type, were incorporated into the pZL1 vector and further utilized for investigation of import characteristics of this dually localized protein. 

In organello import into isolated chloroplasts was performed to investigate chloroplast localization and integration of wt TROL and its presequence mutants into the chloroplast membranes. We expected that some of the amino acid substitutions made to the presequence might interfere with proper processing in the stromal compartment and/or result in alternative localization of TROL. The labeled precursor was imported into organelles and processed into a smaller mature protein of around 66 kDa (Figure 2, mTROL), previously shown to co-purify entirely with the thylakoid fraction [1]. A weaker signal of the size of the labeled precursor was also detected (Figure 2, pTROL). This signal was protected from protease digestion (Figure 2a, lane 5, Figure 2b, lane 2), indicating a portion of TROL located in the IM of chloroplasts, in its non-processed form of 70 kDa [1,5]. In contrast to the wt, the TROL e1 comprised almost entirely of the IM portion, indicating an influence of the 67Ala/67Ile presequence mutation on TROL localization. In this mutant the SPP recognition motif AKS has been changed to IXS, leading to inhibition of stromal processing and the increment of the IM portion of TROL. Compared to wt TROL, it is obvious that for the e1 mutant not only import into thylakoids is impaired, but the IM portion of the protein is highly increased. Since this portion is protected from thermolysin action, as well as from extraction with high salt and carbonate concentrations, we conclude that envelope-TROL is firmly incorporated into the membrane. 

Experiments using increasing external ATP concentrations indicate that TROL requires more than 300 mM ATP for completion of its import into thylakoids, while only 30 mM is necessary for IM incorporation, as visible after thermolysin treatment (Figure 3). This result implies the stop-transfer mechanism of TROL import and its lateral insertion to the IM [11,22]. Time-scale experiments in the presence of 3 mM ATP indicate that the incorporation of TROL into the IM happens very early, and the smaller thylakoid form starts to appear after 2–5 min of import, reaching a maximum between 10–20 min (Figure 4). We observed that after addition of 3 mM ATP in wt, but also in e1, to some extent, there is much less of the IM form of TROL compared to the 300 mM experiment. The distribution of TROL between these two membranes might be influenced by the energy distribution between thylakoids and envelope compartments. TROL could be switching the intensity of its action between the IM (role unknown) and the thylakoids (regulation of photosynthetic electron transfer), influenced/directed by the energetic state of those compartments. 

Jurić et al. [1] have shown that imported TROL could not be extracted from membranes by high salt, urea, or high pH treatments, indicating that At4g01050 is an integral thylakoid membrane protein. The same extraction procedures were used to investigate the membrane incorporation character of the e1–e6 TROL mutants (Figure 5). Only 6 M urea extracted a portion of protein, mainly the IM incorporated one, while TROL in the thylakoids remained fully protected. Mutation e1 causes visible changes in TROL localization, directing most of the protein to the IM of chloroplasts. Once there, the incorporated TROL resists the extraction procedures in the same way as the wild type protein, in which only urea solubilized a portion of the protein from the membranes. After import, the e5 mutation exhibits slightly more IM portion of TROL than the wt, but not significant enough to use it in further experiments.

TROL is not an isolated case in terms of dual localization in chloroplasts. Seventeen other proteins have been identified in both chloroplastic envelope and thylakoid membranes [22]. All of them are predicted to carry an N-terminal signal for chloroplast import. These proteins belong to one of the following groups according to their function: protein transport, tetrapyrrole biosynthesis, membrane dynamics, and transport of nucleotides and inorganic phosphate [22]. One of these proteins is Tic62, a redox sensor in the inner envelope and the thylakoids, proposed to anchor FNR, just like TROL [7].

The nature and the mechanism of dual localization of TROL remains unresolved. The inner envelope of chloroplasts and thylakoid membrane share a similar lipid composition, but perform very different functions. The way in which TROL incorporates into these membranes is at the moment just a speculation. Up to now, no pathway that would catalyse dual targeting has been found. All known nuclear-encoded thylakoid proteins and some IEM proteins are targeted to the respective membranes via a stromal intermediate. TROL, as a dually localized protein, could also use a similar pathway. This has been confirmed by the import properties of the e1 mutant. However, absence of stromal processing for IM located TROL favours the possibility of lateral insertion to the IM. Stromal processing seems to be the key moment for further sorting of TROL to thylakoids. Tha4 and Hcf106 components of the tat pathway are integral membrane proteins that insert into thylakoid membrane by unassisted insertion [25]. The same pathway use PSII subunits W, X, and Y [26], PSI subunit K [27], and the CFoII subunit of the ATP synthase [28], and these have been shown to insert into the membrane by the unassisted pathway. As already mentioned, some IM proteins insert laterally into the membrane by a stop transfer mechanism upon entering the chloroplast. Some of the dual-localized integral proteins, like TROL, may be similarly held at the envelope first, then sorted to the thylakoids. Low ATP demands for insertion of TROL into the IM points to this conclusion. 

Future prospects for the e1 mutant would be the production of transgenic *A. thaliana* plants containing TROL located only/mostly to the IM of chloroplasts. In this way we could study the effect of the absence of TROL from thylakoids on photosynthesis and subsequent electron transfer/dissipation events, as well as the so far unknown role of this protein in the inner envelope membrane.

## 4. Materials and Methods

### 4.1. TROL Presequence Substitutions

Using the QuikChange Multi Site-Directed Mutagenesis method, various mutations were introduced into the TROL presequence. Amino acids 67–78 of the presequence, AKSLTYEEALQQ, represent a partially conserved N-terminal part of the sequence, around the predicted transit peptide cleavage site. In this sequence we have chosen six amino acids potentially significant for TROL processing that might influence its import into and localization inside the chloroplasts. Changes made to the presequence were as following: 

e1: 67Ala→67Ile, e2: 71Thr→71Asn, e3: 72Tyr→72Val, e4:73Glu74Glu→73Gln74Gln, e5: 76Leu→76Thr, e6:78Gln→78Val. Hydrophobicity was checked for each amino acid substitution, according to Kyte and Doolittle [23] (Figure 1). For substitutions 73Glu74Glu→73Gln74Gln, exchange polarity has been compared according to Zimmerman et al. [24] (Figure 1).

Subsequent to the introduction of mutations into the TROL presequence by PCR, constructs were transformed into competent bacteria, multiplied, purified, and checked by restriction enzymes and DNA sequencing. 

### 4.2. In Vitro Transcription and Translation

The coding region for TROL from *Arabidopsis thaliana* was cloned into the vector pZL1 under the control of the T7 promoter and pOE33 from *Pisum sativum* was cloned into the vector pGEM4Z under the control of the SP6 promoter. Transcription and translation were carried out using the TNT^®^ Quick Coupled Transcription/Translation System (Promega, Madison, WI, USA) in the presence of [^35^S]-methionine (185 MBq, PerkinElmer, Boston, MA, USA) for radioactive labelling. After translation, the reaction mixture was centrifuged at 50,000× *g* for 20 min at 4 °C and the post-ribosomal supernatant was used for import experiments. 

### 4.3. Chloroplast Isolation and Protein Import

Chloroplasts were isolated from leaves of 8 days old pea seedlings (*P. sativum* var. Letin, Agricultural Institute Osijek, Osijek, Croatia) and purified through Percoll density gradients as described [11,17]. A standard import reaction contained chloroplasts equivalent to 20 μg chlorophyll in 100 μL import buffer (330 mM sorbitol, 50 mM HEPES/KOH pH 7.6, 3 mM MgSO_4_, 10 mM Met, 10 mM Cys, 20 mM K-gluconate, 10 mM NaHCO_3_, 2% BSA (*w*/*v*)), up to 3 mM ATP and maximal 10% (*v*/*v*) [^35^S]-labeled translation products. Import reactions were initiated by the addition of translation product and carried out for 20 min at 25 °C, unless indicated otherwise. Reactions were terminated by separation of chloroplasts from the reaction mixture by centrifugation through a 40 % (*v*/*v*) Percoll cushion. Chloroplasts were washed once in 330 mM sorbitol, 50 mM HEPES/KOH pH 7.6, and 0.5 mM CaCl_2_, lysed in 10 mM HEPES/KOH pH 7.6 for 30 min on ice and separated into membrane and soluble fractions by centrifugation at 265,000× *g* for 10 min at 4 °C. Import products were separated by SDS–PAGE and radiolabeled proteins analysed by exposure on X-ray films.

For the purpose of investigating the energy requirement, prior to import, ATP was depleted from chloroplasts and the translation product. For chloroplast isolation, plants were taken from the dark and isolated intact chloroplasts were further incubated in the dark on ice for 30 min. to diminish internal ATP production. For the import experiment, 0, 30, 30, and 3000 µM ATP was used and chloroplasts corresponding to 20 µg chlorophyll, in a 20 min import reaction. 

Some experiments included chloroplast protease posttreatment, by using thermolysin after import. Thermolysin in concentration of 0.5 µg per µg chlorophyll was applied for 20 min on ice. The reaction was stopped by adding 5 mM EDTA. Chloroplasts were pelleted and resuspended in Laemmli buffer [29].

### 4.4. Membrane Extraction of Imported Proteins

Subsequent to import of 20 min at 25 °C, chloroplasts were re-isolated, washed, and separated to the membrane and soluble fractions. Isolated membranes, containing imported proteins, were treated with 6 M Urea in 10 mM HEPES/KOH pH 7.6, 0.1 M Na_2_CO_3_ pH 11.5, or 1 M NaCl. All incubations were performed for 20 min on RT. As a control, membranes were incubated solely in 10 mM HEPES/KOH pH 7.6 for 30 min on ice. Afterwards, samples were centrifuged at 265,000× *g* for 10 min. at 4 °C, and both pellets and the supernatants were analysed by SDS–PAGE and by the exposure on X-ray films.

## Figures and Tables

**Table 1:** Mutations introduced to the TROL presequence by the QuikChange Multi Site-Directed Mutagenesis and corresponding primer sequences.

Change	Mutation/Primer Name	Primer Sequence
e1	g199a_c200t	aagcagtgcaacagctcctattaaatccctgacgtacgag
e2	c212a_g213t	gctcctgctaaatccctgaattacgaggaagctctgcaac
e3	t214g_a215t	ctgctaaatccctgacggtcgaggaagctctgcaac
e4	g217c_g220c	gctaaatccctgacgtaccagcaagctctgcaacaatcta
e5	c226a_t227c	ccctgacgtacgaggaagctacgcaacaatctatgacca
e6	c232g_a233t	cgtacgaggaagctctgcaagtatctatgaccacttcttca
	TROLfor	caccatggaagctctgaaaaccgca
	TROLrev	gggctgcgatggcatcg

## Abbreviations

TROL	thylakoid rhodanase-like protein
FNR	ferredoxin:NADP^+^ oxidoreductase
RHO	rhodanase-like domain
ITEP	highly conserved module of TROL necessary for establishing high-affinity interaction with FNR
PEPE	Pro-Val-Pro repeat-rich region
IM	inner envelope membrane
SPP	stromal processing peptidase
